# Extracellular HMGB1 promotes CD44 expression in hepatocellular carcinoma via regulating miR-21

**DOI:** 10.18632/aging.202649

**Published:** 2021-03-04

**Authors:** Jun Li, Haozhen Ren, Jinglin Wang, Pengfei Zhang, Xiaolei Shi

**Affiliations:** 1 Department of Hepatobiliary Surgery, Affiliated Drum Tower Hospital of Nanjing University Medical School, Nanjing 210008, Jiangsu Province, China

**Keywords:** extracellular HMGB1, hepatocellular carcinoma, CD44, miR-21

## Abstract

As a member of damage-associated molecular patterns (DAMPs), extracellular high-mobility group box 1 (HMGB1) plays a critical role in hepatocellular carcinoma (HCC) progression. Cluster differentiation 44 (CD44) has been demonstrated to participate in HCC progression. However, the relationship between extracellular HMGB1 and CD44 remains unclear. In this study, our results indicated that extracellular HMGB1 promoted the invasion, sphere formation and EMT process of HCC by increasing CD44 expression, which was dependent on miR-21. Moreover, miR-21 upregulated CD44 expression via activating OCT4/TGF-β1 signaling. Finally, we demonstrated the activation of Rage/JNK signaling caused by extracellular HMGB1 was responsible for miR-21 overexpression. Together, these findings reveal an important role of extracellular HMGB1 in HCC progression through upregulating miR-21/CD44.

## INTRODUCTION

Hepatocellular carcinoma (HCC) is the fifth most common malignancy worldwide [1, 2]. Most cases of HCC are developed from chronic liver diseases, such as hepatic B or C virus infection, alcoholic liver disease and nonalcoholic steatohepatitis [1, 2]. Considering the high rate of metastasis and postsurgical recurrence, the prognoses of HCC patients are extremely poor and the 5-year survival rate is only 18% [1, 2]. Therefore, it is important to explore the molecular mechanisms of HCC progression.

Many inflammatory factors such as interleukin-1beta (IL1b), interleukin-17a (IL17a) and interleukin-6 (IL6) have been reported to facilitate HCC progression by promoting proliferation, metastasis and cancer stem cells (CSCs) formation [3, 4]. Intracellular high-mobility group box 1 (HMGB1) is a conserved chromatin-binding protein and maintains genome stability [5]. Extracellular HMGB1 could be passively released from necrotic cells and actively excreted by inflammatory cells or tumor cells [6]. Accumulating data have revealed that HMGB1 plays a complicated and critical role in various diseases including HCC [7–9]. The receptor for Advanced Glycation End Products (Rage) and Toll-like receptors (TLRs), such as TLR2, TLR4 and TLR9, are verified as the receptors of extracellular HMGB1 [6]. With the interactions to these receptors, extracellular HMGB1 acts as an inflammatory mediator and contributes to tumor metastasis, angiogenesis and chemotherapy resistance [10, 11].

CD44 is one of the most important CSCs markers [12]. CSCs are small subpopulations of tumor cells and characterized with stem cell-like properties, such as self-renewal [13]. Mounting evidence has revealed CD44 promotes tumor metastasis and contributes to therapeutic resistance [14–16]. However, the relationship between extracellular HMGB1 and CD44 remains unclear.

MicroRNAs are single-strand and small (21-25 nucleotides) RNAs, which regulate relevant gene expressions at posttranslational level. Alternations of microRNAs are tightly associated with tumor progression [17, 18]. Accumulating data has indicated miR-21 leads to the development and progression of diverse tumors including HCC [19–22]. Recently, Chen et al indicate that extracellular HMGB1 promotes HCC metastasis via miR-21 mediated upregulation of MMPs [23]. Additionally, hyaluronic acid (HA) has been reported to increase miR-21 expression through activating CD44/ JNK/c-Jun pathway [24]. JNK/c-Jun pathway belongs to MAPK signaling pathway, which is the main downstream of extracellular HMGB1 [9]. Therefore, it seems that miR-21 plays a critical role in extracellular HMGB1-mediated HCC progression.

In this study, we investigated a critical role of extracellular HMGB1 in HCC progression via increasing miR-21/CD44 axis and confirmed that miR-21 upregulated CD44 expression through OCT4/TGF-β1 signaling. Moreover, we found activation of Rage/JNK signaling was essential for miR-21 expression caused by extracellular HMGB1. Taken together, our findings suggest a new molecular basis of extracellular HMGB1 mediated HCC progression by increasing CD44, which may provide new targets and strategies for HCC treatment.

## RESULTS

### Extracellular HMGB1 is positively associated with CD44 expression

**Figure 1:**
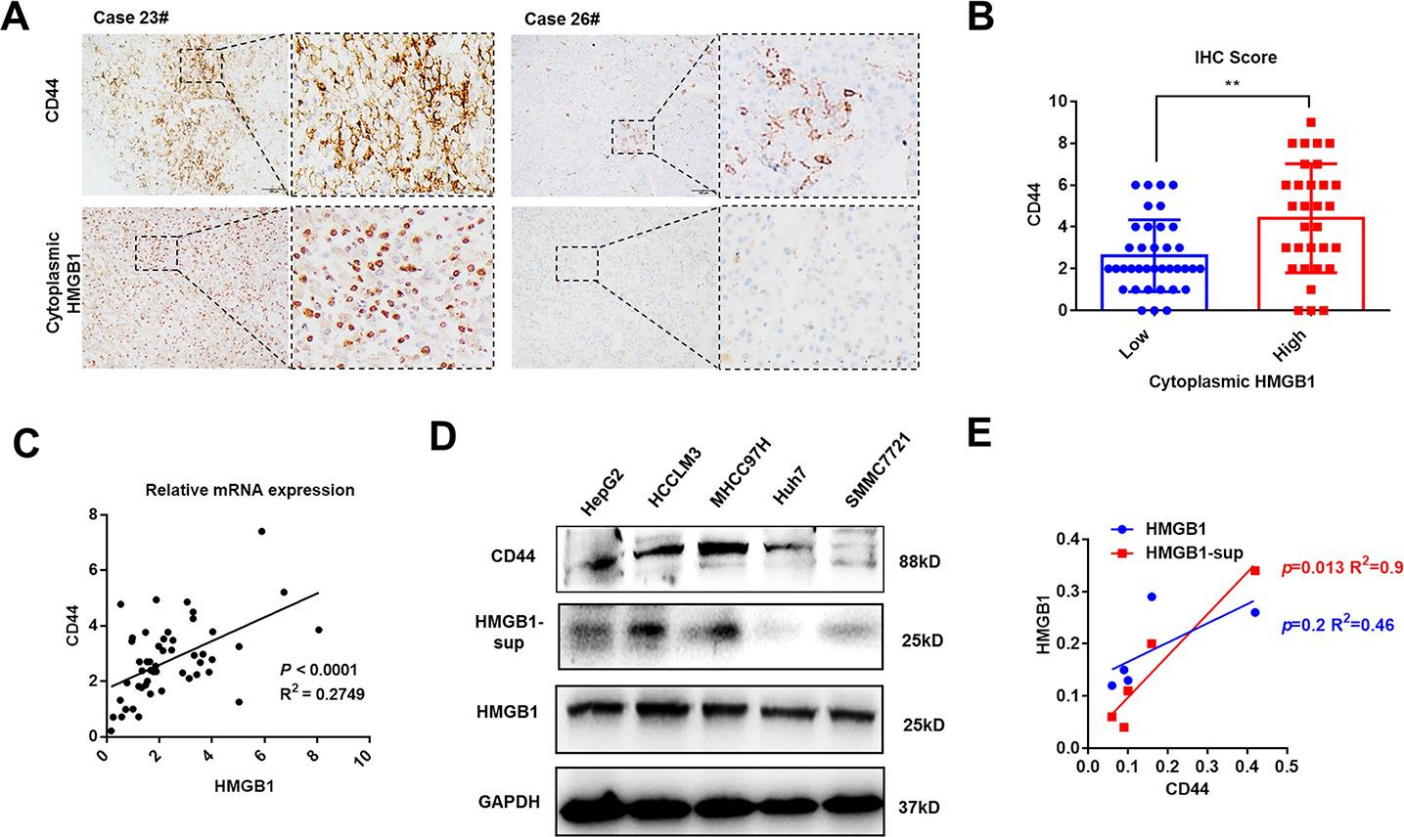
**HMGB1 is positively associated with CD44 in HCC.** (**A**) Representative images show different intensities of staining CD44 and cytoplasmic HMGB1 in HCC samples. Scale bars, 100um. (**B**) HCC Patients with high expression of cytoplasmic HMGB1 are characterized by high expression of CD44 through analyzing IHC score. n=68. (**C**) Positive correlation of CD44 and HMGB1 in HCC samples by analyzing the mRNA levels. n=50. (**D**, **E**) Different expression of CD44, HMGB1 and supernatant HMGB1 (HMGB1-sup) are measured in five HCC cell lines via immunoblot analysis and results are quantified. HMGB1-sup rather than HMGB1 is positively correlated with CD44. Data are means ± SEM, * means p<0.05, ** means p<0.01, *** means p<0.001 by unpaired student T test.

Mounting evidence has revealed the crucial role of HMGB1 in tumor progression [9]. As shown in Figure 1A and Supplementary Figure 1A, HMGB1 was mostly expressed in tumor cells and the intensity of cytoplasmic HMGB1 varied in different HCC patients. To investigate the relationship between cytoplasmic HMGB1 and CD44, we performed immunohistochemistry experiments in 68 HCC tissues and demonstrated a tight association between cytoplasmic HMGB1 and CD44 (Figure 1A, 1B), which indicated there was a positive correlation between extracellular HMGB1 and CD44. The clinic pathologic characteristics of cytoplasmic HMGB1 and CD44 in 68 cases of HCC were summarized in Table 1. Both of CD44 and cytoplasmic HMGB1 correlated to poor prognosis. In order to explore the correlation of HMGB1 and CD44, cellular RNA was collected from 50 HCC tissues and used for Q-PCR analysis. As shown in Figure 1C, HMGB1 closely related to CD44 at mRNA level. Besides, we detected the protein level of CD44 and HMGB1 in five HCC cell lines. Interestingly, compared to intracellular HMGB1, HMGB1-sup, namely extracellular HMGB1, was positively associated to CD44 expression (Figure 1D, 1E). Moreover, we observed that concentration of serum HMGB1 from HCC patients was positively associated with CD44 IHC score (Supplementary Figure 1C). Together, these findings indicated HMGB1, especially extracellular HMGB1, positively correlated to CD44 in HCC samples and cell lines.

**Table 1:** The clinicopathologic characteristics of 68 cases of HCC.

	**CD44**			**Cytoplasmic HMGB1**		
	**Low**	**High**		**Low**	**High**	
**n(%)**	40(58.8)	28(41.2)	*P* value	37(54.4)	31(45.6)	*P* value
**Gender**						
Male	23(33.8)	25(36.8)	0.190	20(29.4)	28(41.2)	0.530
Female	13(19.1)	7(10.3)		10(14.7)	10(14.7)	
**Age, y**						
≤50	21(30.9)	18(26.5)	0.910	22(32.4)	27(39.7)	0.560
>50	16(23.5)	13(19.1)		15(22.1)	14(20.6)	
**HBsAg**						
Negative	14(20.6)	8(11.8)	0.120	17(25.0)	5(7.3)	0.014
Positive	20(29.4)	26(38.2)		21(30.9)	25(36.8)	
**AFP, ng/ml**						
≤20	28(41.2)	12(17.6)	0.051	30(44.1)	10(14.7)	0.003
>20	13(19.1)	15(22.1)		11(16.2)	17(25.0)	
**Cirrhosis**						
No	15(22.1)	16(23.5)	0.020	20(29.4)	11(16.2)	0.670
Yes	28(41.2)	9(13.2)		22(32.4)	15(22.0)	
**Tumor size, cm**						
≤5	21(30.9)	9(13.2)	0.560	23(33.8)	7(10.3)	0.230
>5	29(42.7)	9(13.2)		24(35.3)	14(20.6)	
**Tumor number**						
Single	34(50.0)	11(16.2)	0.369	32(47.1)	13(19.1)	0.527
Multiple	15(22.1)	8(11.7)		18(26.5)	5(7.3)	
**Vascular invasion**						
No	38(55.9)	12(17.6)	0.041	35(51.5)	15(22.1)	0.054
Yes	9(13.2)	9(`3.2)		8(11.7)	10(14.7)	
**TNM stage**						
I-II	34(50.0)	12(17.6)	0.003	33(48.5)	13(19.1)	0.035
III-IV	8(11.7)	14(20.7)		10(14.7)	12(17.7)	

Abbreviations: AFP: alpha fetoprotein; HBsAg: hepatitis B surface antigen.

### Extracellular HMGB1 promotes HCC progression via increasing CD44

**Figure 2:**
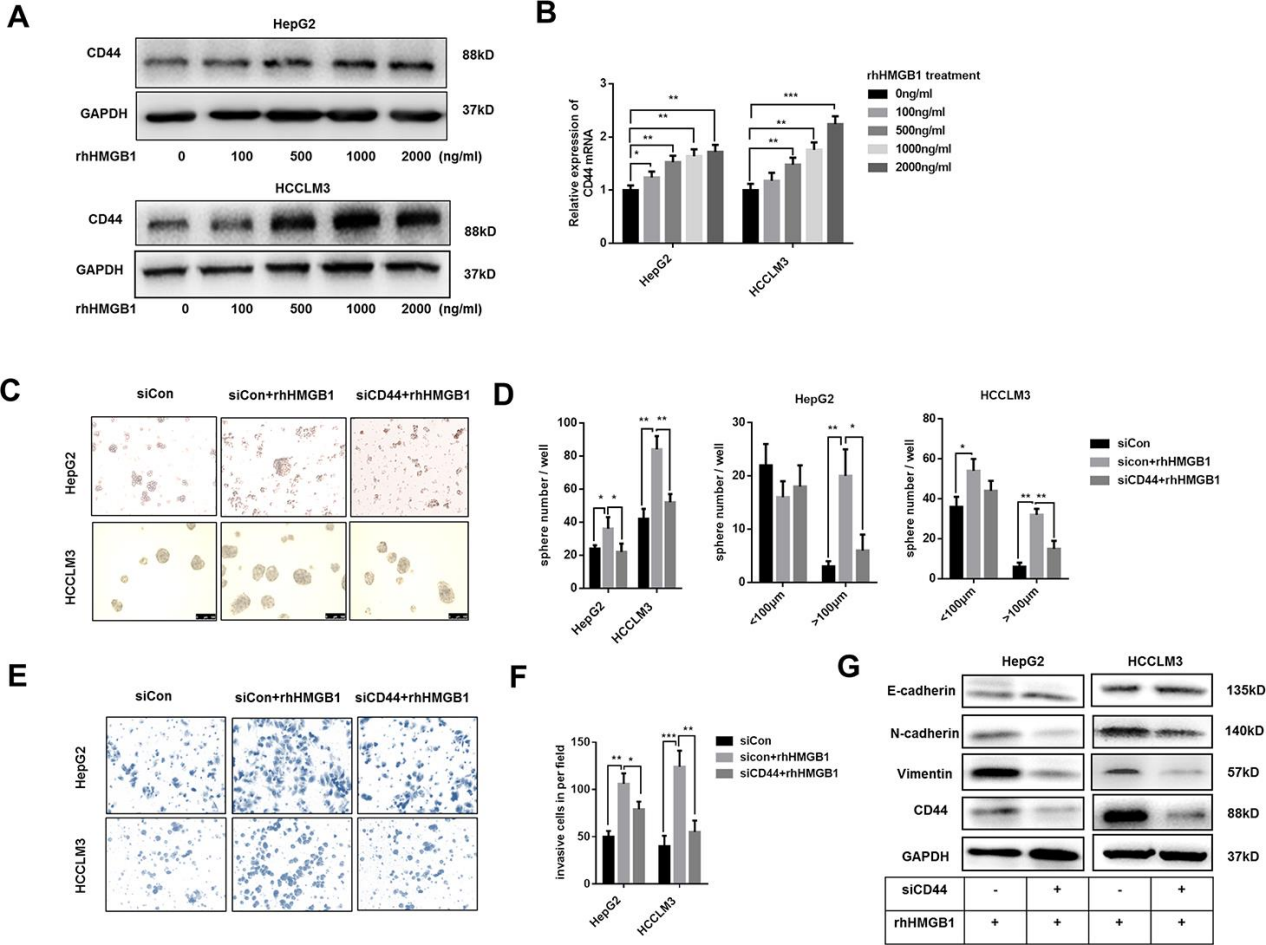
**Extracellular HMGB1 promotes the sphere formation, invasion and EMT process in CD44-dependent way.** (**A**) Immunoblot analysis shows rhHMGB1 promotes CD44 expression in a dose-dependent manner. HepG2 and HCCLM3 cells were cultured with different concentration of rhHMGB1 for 24h. (**B**) Q-PCR analysis shows that CD44 is upregulated by rhHMGB1 in a dose-dependent manner. (**C**, **D**) rhHMGB1 treatment promotes HCC sphere formations in CD44-dependent way. Number and size of spheres were photographed and analyzed. HepG2 and HCCLM3 cells were transfected with negative siRNA or CD44 siRNA and then cultured with rhHMGB1 (1μg/ml) for 24h. (**E**, **F**) Invasion experiments show rhHMGB1 promotes invasive abilities of HCC cells in CD44-dependent way. The invaded cells were counted and results were analyzed. HepG2 and HCCLM3 cells were transfected with negative siRNA or CD44 siRNA and then cultured with rhHMGB1 (1μg/ml) for 24h. (**G**) Immunoblot analysis shows that targeting CD44 inhibits EMT process caused by rhHMGB1. HepG2 and HCCLM3 cells were transfected with negative siRNA or CD44 siRNA and then cultured with rhHMGB1 (1μg/ml) for 24h. Data are means ± SEM, * means p<0.05, ** means p<0.01, *** means p<0.001 by unpaired student T test.

Extracellular HMGB1 could be actively or passively released from cell cytoplasm [6]. To further probe into the relationship between extracellular HMGB1 and CD44, HepG2 and HCCLM3 cells were cultured with different concentration of recombinant human HMGB1 (rhHMGB1) for 24h. As shown in Figure 2A, extracellular HMGB1 increased CD44 expression in a dose-dependent manner. Additionally, the mRNA level of CD44 was also increased by rhHMGB1 (Figure 2B). Interestingly, CSCs markers such as CD133, EpCAM and OCT4 were all strengthened by rhHMGB1 (Supplementary Figure 2A), which uncovered an important role of extracellular HMGB1 in CSCs formation.

Both extracellular HMGB1 and CD44 are reported to facilitate HCC progression [23]. To further explore the relationship between extracellular HMGB1 and CD44, HepG2 and HCCLM3 were cultured in serum-free medium with or without rhHMGB1 for sphere formation assay. As shown in Figure 2C, 2D, CD44 knockdown by CD44 siRNA decreased the total number and size of tumor spheres stimulated by rhHMGB1. Moreover, rhHMGB1 mediated invasion and migration capacities were both repressed by CD44 deficiency (Figure 2E, 2F and Supplementary Figure 2B). Considering roles of HMGB1 and CD44 in EMT process in varied tumors, we observed that CD44 knockdown contributed to the reversal of EMT process caused by rhHMGB1 (Figure 2G). Taken together, our findings suggested that extracellular HMGB1 increased CD44 expression and strengthened HCC progression in CD44-dependent way.

### miR-21 is essential for extracellular HMGB1 mediated CD44 expression

**Figure 3:**
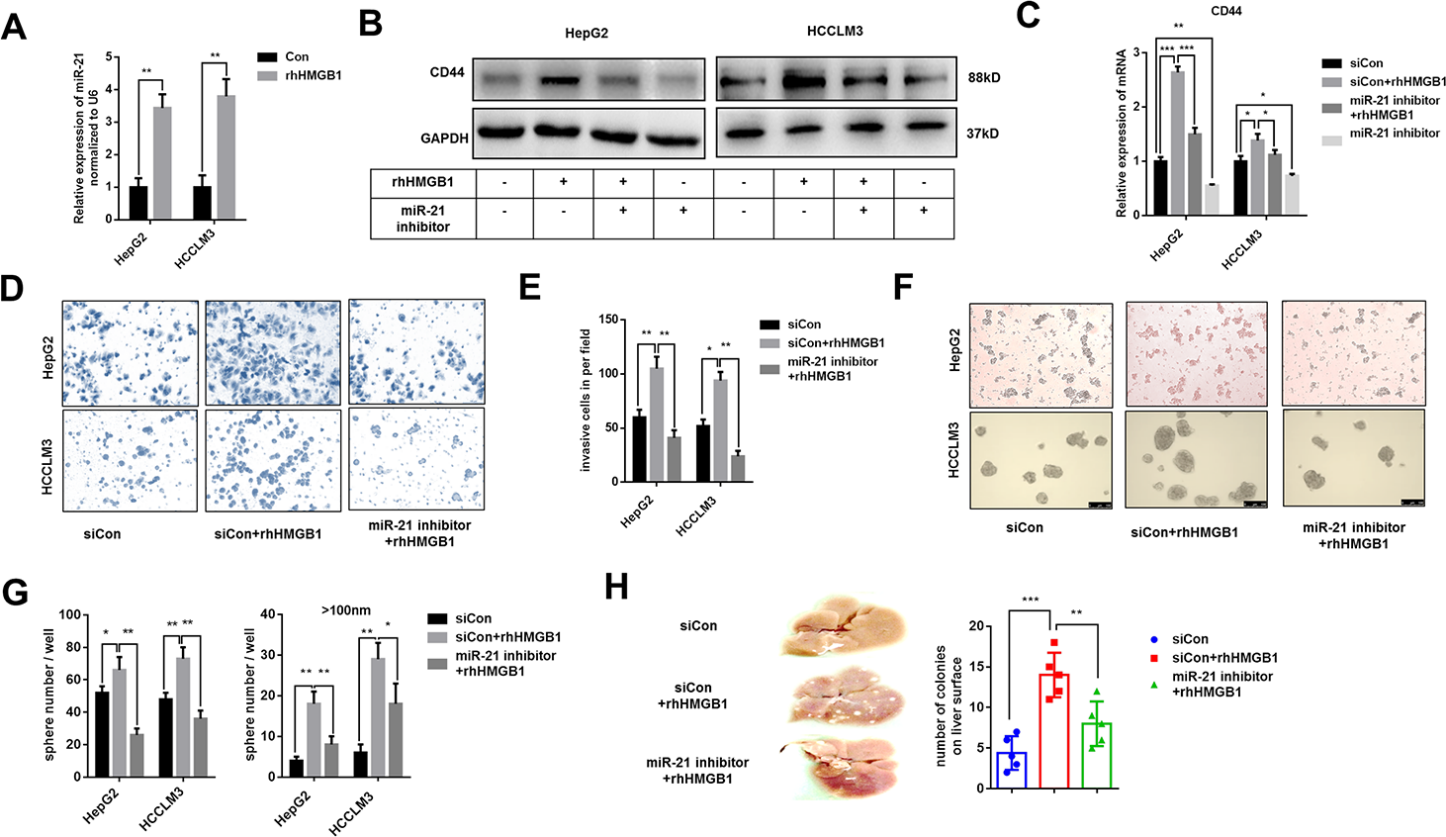
**Extracellular HMGB1 increases CD44 expression via upregulating miR-21.** (**A**) Q-PCR analysis shows that rhHMGB1 increases miR-21 expression. HepG2 and HCCLM3 cells were cultured with rhHMGB1 (1μg/ml) for 24h. (**B**) Immunoblot analysis shows that miR-21 inhibitor represses CD44 expression caused by rhHMGB1. HepG2 and HCCLM3 cells were transfected with negative control or miR-21 inhibitor and then cultured with rhHMGB1 (1μg/ml) for 24h. (**C**) Q-PCR analysis indicates that miR-21 inhibitor represses CD44 expression caused by rhHMGB1. HepG2 and HCCLM3 cells were transfected with negative control or miR-21 inhibitor and then cultured with rhHMGB1 (1μg/ml) for 24h. (**D**, **E**) Results from Invasion experiments indicate miR-21 inhibitor represses HCC invasion caused by rhHMGB1. HepG2 and HCCLM3 cells were transfected with negative control or miR-21 inhibitor and then cultured with rhHMGB1 (1μg/ml) for 24h. (**F**–**H**) Results from sphere formations experiments indicate miR-21 inhibitor represses HCC sphere formations caused by rhHMGB1. HepG2 and HCCLM3 cells were transfected with negative control or miR-21 inhibitor and then cultured with rhHMGB1 (1μg/ml) for 24h. (**G**) Results from metastasis model *in vivo* show that miR-21 inhibitor suppresses liver metastasis caused by rhHMGB1. HepG2 cells were transfected with negative control or miR-21 inhibitor and then cultured with rhHMGB1 (1μg/ml) for 24h. Data are means ± SEM, * means p<0.05, ** means p<0.01, *** means p<0.001 by unpaired student T test.

miR-21 is reported to promote HCC progression and could be increased by extracellular HMGB1 [23]. Several studies have demonstrated a crucial role of miR-21 in CSCs formation of colon cancer and breast cancer [25–27]. Through analyzing public data, we found miR-21 positively associated to CD44 in HCC (Supplementary Figure 3A). To explore the relationship between miR-21 and CD44, HepG2 cells were transfected with miR-21 inhibitor or miR-21 mimic respectively. As shown in Supplementary Figure 3B, 3C, miR-21 regulated CD44 expression both in mRNA and protein level. We confirmed that miR-21 expression was obviously enhanced by rhHMGB1 as reportedly (Figure 3A). To investigate the relationship between miR-21 and CD44 in rhHMGB1-treated cells, miR-21 inhibitor was used to silence miR-21 expression. We found that miR-21 inhibitor downregulated CD44 expression stimulated by rhHMGB1 (Figure 3B, 3C, Supplementary Figure 3D). Moreover, miR-21 inhibitor significantly suppressed the capacities of sphere formation and invasion caused by rhHMGB1 (Figure 3D–3G). We also performed the metastatic model *in vivo* and observed that miR-21 inhibitor efficiently decreased the number of tumor colonies on liver surface and lung caused by rhHMGB1 (Figure 3H and Supplementary Figure 3E). Collectively, these observations indicated that miR-21 was essential for rhHMGB1 mediated HCC progression by modulating CD44 expression.

### miR-21 increases CD44 expression by activating OCT4/TGF-β1 axis

**Figure 4:**
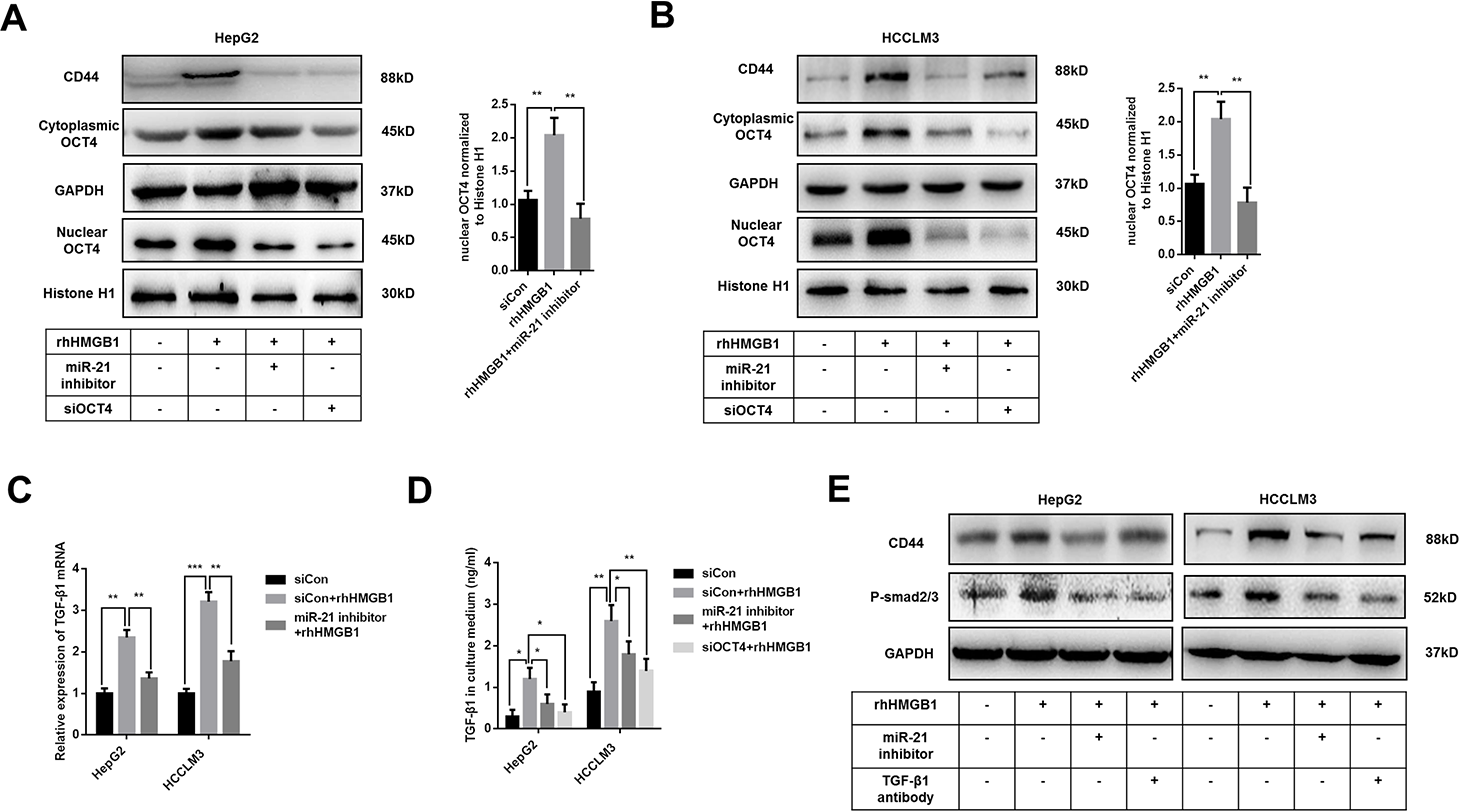
**Extracellular HMGB1 promotes CD44 expression through miR-21 mediated activation of OCT4/ TGF-β1 signaling.** (**A**, **B**) Immunoblot analysis shows that miR-21 inhibitor restricts OCT4 expression and nuclear translocation caused by rhHMGB1. HepG2 and HCCLM3 cells were transfected with negative control, OCT4 siRNA or miR-21 inhibitor and then cultured with rhHMGB1 (1μg/ml) for 24h. (**C**) Q-PCR analysis indicates miR-21 inhibitor represses TGF-β1 expression caused by rhHMGB1. HepG2 and HCCLM3 cells were transfected with negative control or miR-21 inhibitor and then cultured with rhHMGB1 (1μg/ml) for 24h. (**D**) Supernatant TGF-β1 is measured by ELISA assays and results indicate that miR-21 inhibitor and OCT4 siRNA both suppresses TGF-β1 secretion caused by rhHMGB1. HepG2 and HCCLM3 cells were transfected with negative control, OCT4 siRNA or miR-21 inhibitor and then cultured with rhHMGB1 (1μg/ml) for 24h. (**E**) Immunoblot analysis shows that miR-21 inhibitor and TGF-β1 antibody both suppress CD44 expression by inactivating TGF-β1/Smad signaling. HepG2 and HCCLM3 cells were treated with negative control, TGF-β1 antibody or miR-21 inhibitor and then cultured with rhHMGB1 (1μg/ml) for 24h. Data are means ± SEM, * means p<0.05, ** means p<0.01, *** means p<0.001 by unpaired student T test.

Previous results indicated that rhHMGB1 promoted OCT4 mRNA expression (Supplementary Figure 2A). We also found miR-21 was significantly associated with OCT4 expression by analyzing public data (Supplementary Figure 4A). Meanwhile, miR-21 inhibitor suppressed OCT4 mRNA expression caused by rhHMGB1, which indicated the essential role of miR-21 in rhHMGB1 mediated OCT4 expression (Supplementary Figure 4B). In rhHMGB1-treated HCC cells, we also observed that miR-21 inhibitor abrogated both OCT4 protein expression and its nuclear translocation (Figure 4A, 4B). Immunofluorescence experiments staining OCT4 were performed and results confirmed that rhHMGB1 mediated OCT4 nuclear transport was repressed by miR-21 inhibitor (Supplementary Figure 4C). Additionally, miR-21 inhibitor and OCT4 knockdown both significantly decreased CD44 expression caused by rhHMGB1, which indicated miR-21 upregulated CD44 via OCT4 (Figure 4A, 4B).

As shown in Figure 4C, 4D, mRNA and protein level of TGF-β1 were upregulated by rhHMGB1. Both miR-21 inhibitor and OCT4 siRNA efficiently suppressed TGF-β1 expression caused by rhHMGB1, which suggested rhHMGB1 promoted TGF-β1 through miR-21/OCT4 axis (Figure 4C, 4D). Moreover, rhHMGB1 activated TGF-β1/Smad signaling via miR-21 (Figure 4E). Lastly, TGF-β1 neutralizing antibody suppressed CD44 expression caused by rhHMGB1, suggesting autocrine TGF-β1/Smad signaling was essential for CD44 overexpression (Figure 4E).

Collectively, these findings indicated that miR-21 mediated OCT4/TGF-β1 axis was responsible for CD44 expression in rhHMGB1-treated cells.

### Extracellular HMGB1 promotes CD44 expression via activating Rage/JNK signaling

**Figure 5:**
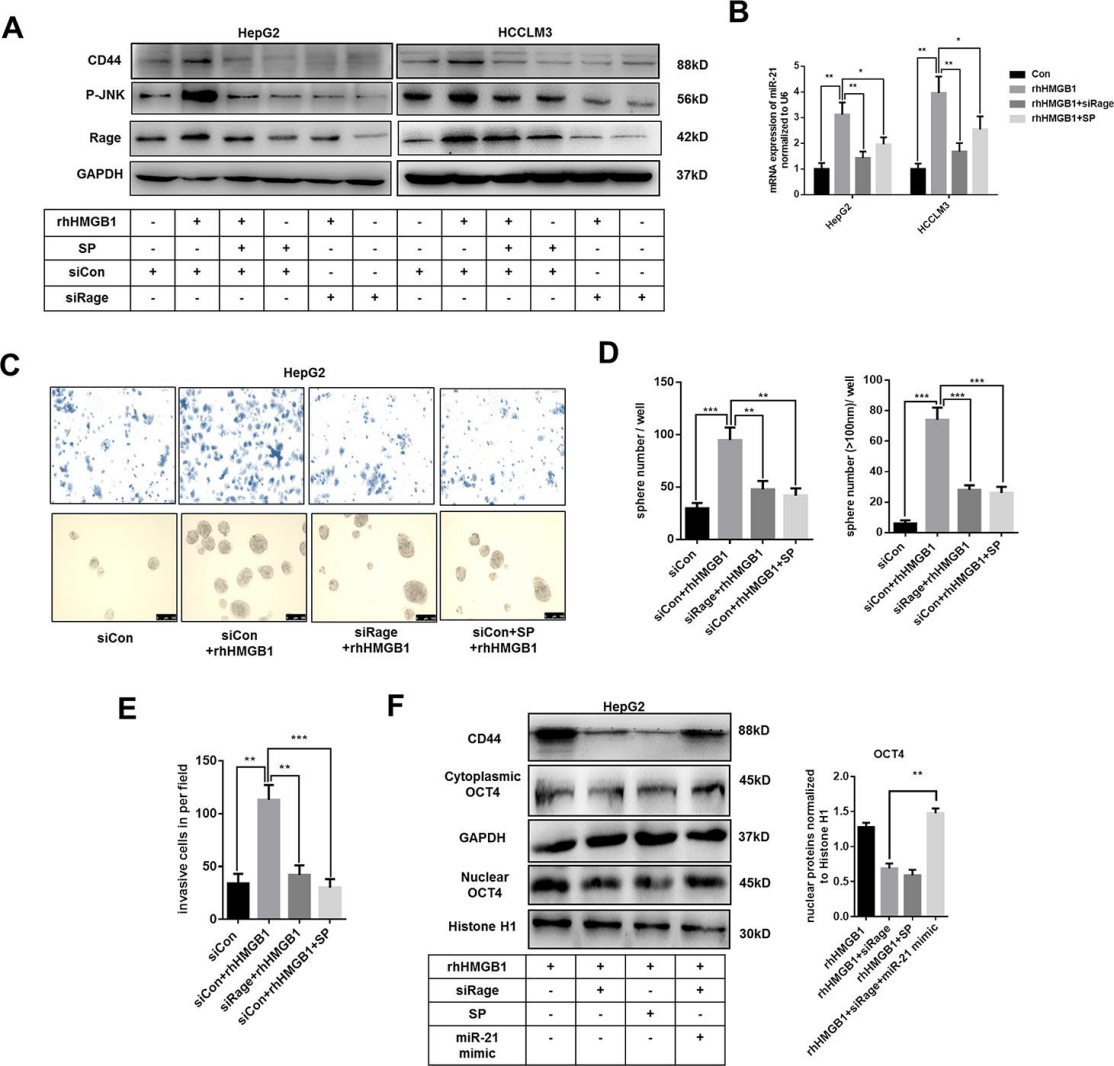
**Extracellular HMGB1 upregulates miR-21 expression via activating Rage/JNK signaling pathway.** (**A**) Immunoblot analysis indicates rhHMGB1 promotes CD44 expression by activating Rage/JNK signaling pathway. HepG2 and HCCLM3 cells were treated with negative control, Rage siRNA, JNK inhibitor(SP600125,SP,20μm) and rhHMGB1 (1μg/ml) for 24h. (**B**) Q-PCR analysis indicates inactivating Rage/JNK signaling downregulates miR-21 expression caused by rhHMGB1. HepG2 and HCCLM3 cells were treated with negative control, Rage siRNA, SP(20μm) and rhHMGB1 (1μg/ml) for 24h. (**C**–**E**) Rage/JNK pathway accounts for HCC sphere formations and invasion caused by rhHMGB1. Number and size of spheres and invaded cells were photographed and analyzed. HepG2 cells were treated with negative control, Rage siRNA, SP(20μm) and rhHMGB1 (1μg/ml) for 24h. (**F**) Immunoblot analysis indicates miR-21 mimic, Rage siRNA and SP all decrease CD44 expression and OCT4 nuclear translocation caused by rhHMGB1. HepG2 cells were treated with negative control, Rage siRNA, SP(20μm), miR-21 mimic and rhHMGB1 (1μg/ml) for 24h. Data are means ± SEM, * means p<0.05, ** means p<0.01, *** means p<0.001 by unpaired student T test.

Rage is involved in inflammatory diseases and associated with HCC progression [28, 29]. JNK pathway was downstream of Rage signaling [28]. As shown in Figure 5A and Supplementary Figure 5A, rhHMGB1 significantly activated Rage/JNK signaling. To explore the role of Rage/JNK signaling in rhHMGB1-mediated CD44 expression, Rage siRNA and JNK inhibitor, SP600125 (SP), were used to inactivate Rage/JNK signaling. We observed that Rage siRNA or SP both suppressed CD44 expression induced by rhHMGB1 (Figure 5A and Supplementary Figure 5A), which suggested Rage/JNK signaling was essential for CD44 expression. Moreover, Rage siRNA or SP significantly inhibited miR-21 expression induced by rhHMGB1 (Figure 5B). These findings suggested rhHMGB1 increased expressions of miR-21 and CD44 via activating Rage/JNK signaling.

Transwell experiments and sphere formation assays were performed to evaluate the effects of Rage/JNK signaling exerted in HCC progression caused by rhHMGB1. Rage siRNA and SP abrogated the increased capacities of invasion and sphere formation in rhHMGB1-treated cells (Figure 5C–5E). Moreover, Rage siRNA or SP effectively suppressed OCT4 expression and its nuclear translocation (Figure 5F). In rhHMGB1-treated cells, miR-21 mimic reactivated OCT4/CD44 expression in the absence of Rage, which further confirmed that miR-21/OCT4/CD44 axis was downstream of Rage signaling (Figure 5F). Collectively, these findings revealed that Rage/JNK signaling pathway accounted for the upregulation of miR-21/CD44 caused by extracellular HMGB1.

### Correlation of related proteins in HCC tissues and patient prognosis

**Figure 6:**
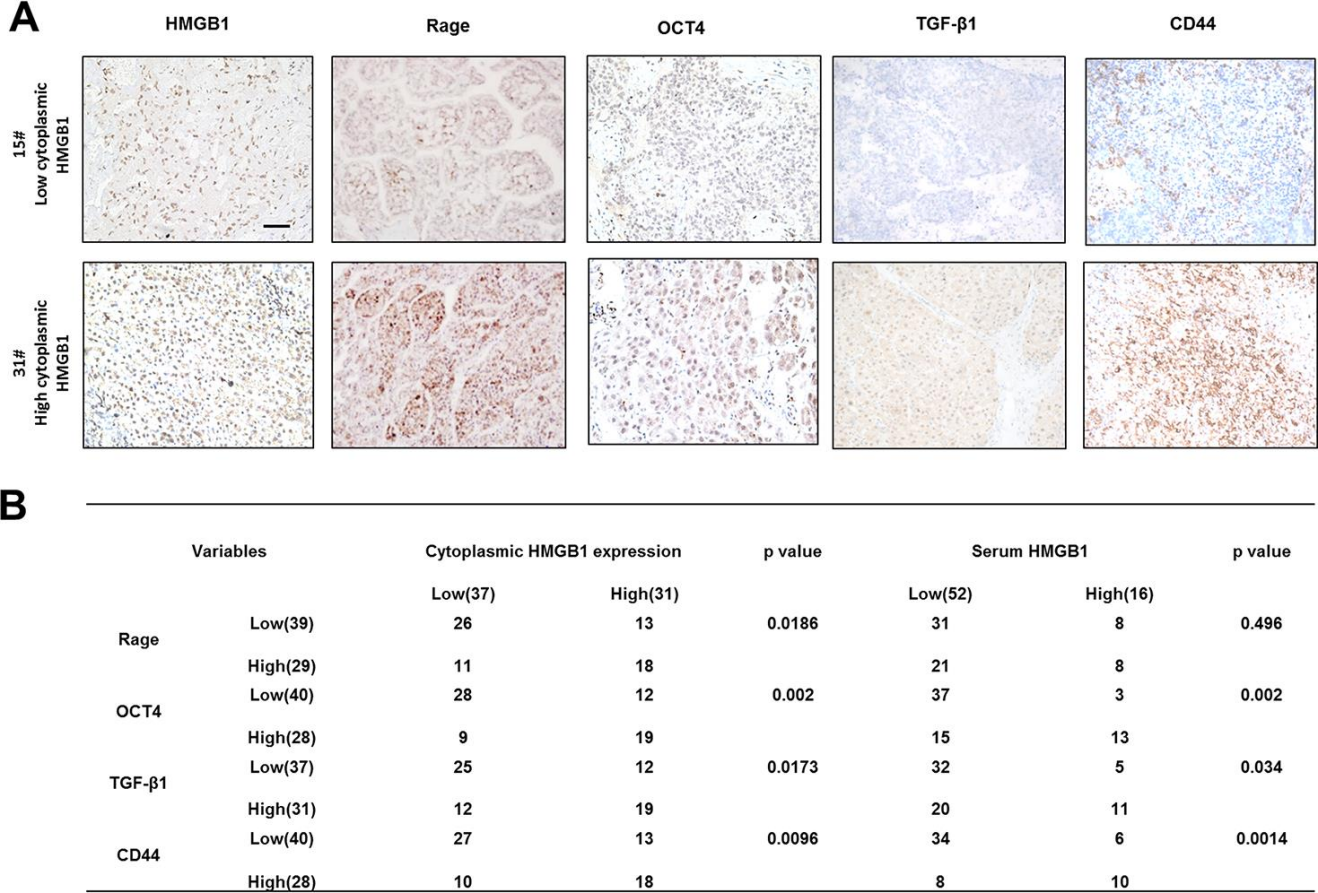
**Correlation of related proteins in clinical HCC samples.** (**A**) Representative images staining HMGB1, Rage, OCT4, TGF-β1 and CD44 in HCC samples. Scale bars, 100um. (**B**) Correlation of related proteins and cytoplasmic or serum HMGB1 in HCC samples. n=68. Data are means ± SEM, * means p<0.05, ** means p<0.01, *** means p<0.001 by unpaired student T test.

To explore the relationship between cytoplasmic HMGB1 and related proteins, we performed IHC staining HMGB1, Rage, OCT4/TGF-β1 axis and CD44 in HCC patients (Figure 6A). In accordance with former studies, HCC patients with higher expression of cytoplasmic HMGB1 or serum HMGB1 were characterized with higher expression of Rage, CD44 and OCT4/TGF-β1 axis (Figure 6B). These data further demonstrated the positive relationship between extracellular HMGB1 and CD44.

## DISCUSSION

Recent studies indicated CD44 was highly related to poor prognosis of HCC and responsible for tumor progression [30–33]. As the special receptor for HA, most effects caused by HA were dependent on CD44 [34]. Accumulating data show that CD44 promotes tumor progression via regulating proliferation and EMT process [30]. However, the underlying mechanism of CD44 overexpression in HCC remains unclear.

In this present study, we found extracellular HMGB1 instead of intracellular HMGB1 positively associated with CD44 expression. HMGB1 is considered as an inflammatory mediator involved in various diseases including tumors [35]. Recent studies show high expression of HMGB1 is strongly related to poor prognosis of HCC patients [9, 11]. We observed that rhHMGB1 promoted the sphere formation, invasion and EMT process of HCC cells. Moreover, targeting CD44 could abolish rhHMGB1-induced HCC progression. These data suggested extracellular HMGB1 might participate in CD44 overexpression, which in turn was essential for extracellular HMGB1-mediated tumor promotion.

miR-21 is significantly associated with extracellular HMGB1 [23]. Our data verified rhHMGB1 increased CD44 expression by upregulating miR-21. With the treatment of miR-21 mimic or inhibitor, we demonstrated that miR-21 upregulated CD44 expression. Results from transwell and sphere formation experiments revealed the critical role of miR-21 in rhHMGB1-induced CD44 overexpression. Additionally, we demonstrated that miR-21 promoted CD44 expression via activating OCT4/TGF-β1 axis in HCC cells cultured with rhHMGB1. Although we confirmed that miR-21 was indispensable for the activation of OCT4/TGF-β1 axis, especially for OCT4 expression and nuclear translocation, the detailed molecular basis remains unclear and more efforts are needed in the future.

Rage is one receptor for HMGB1 [6, 28]. After interacting to Rage, many intracellular signaling pathways are activated such as NF-κB, PI3K/AKT and MAPK. Recently, Rage is reported to promote HCC progression by inhibiting autophagy [29]. In our study, Rage siRNA or JNK inhibitor SP600125 abrogated miR-21 expression caused by rhHMGB1, suggesting Rage/JNK signaling accounted for miR-21 induction.

Moreover, invasion capacity and sphere formation caused by rhHMGB1 were both also repressed by Rage siRNA or SP600125, which documented that Rage/JNK signaling was essential for extracellular HMGB1 mediated HCC progression.

Taken together, our study confirmed the central role of miR-21 in extracellular HMGB1 mediated CD44 expression. Firstly, CD44 is essential for HCC invasion and sphere formation caused by extracellular HMGB1. Secondly, extracellular HMGB1 promoted CD44 expression via activating miR-21/OCT4/TGF-β1 axis. Lastly, we demonstrated that Rage/JNK signaling activated by extracellular HMGB1 was responsible for miR-21 upregulation. These findings confirmed the importance of extracellular HMGB1 in HCC progression by activating Rage/miR-21/OCT4/CD44 cascades and indicated extracellular HMGB1 as a potential anticancer target.

## MATERIALS AND METHODS

### Patients and specimens

Samples were achieved from 68 HCC patients who had undergone curative resection between 2014 and 2016 and were pathologically confirmed HCC at Medical School of Nanjing University Affiliated Drum Tower Hospital. This project was approved by the Ethical Committee of Medical School of Nanjing University Affiliated Drum Tower Hospital. Informed consent for the present study was received from all patients prior to the commencement of the experiments. The clinical signatures of all patients are summarized in Supplementary Table 1.

### Animals and chemical reagents

Male BALB/c nu/nu mice (6-8 weeks old, Shanghai Institute of Material Medicine, Chinese Academy of Science) were housed in specific pathogen-free conditions. All animals received humane care according to the criteria outlined in the "Guide for the Care and Use of Laboratory Animals" prepared by the National Academy of Sciences and published by the National Institutes of Health (NIH publication 86-23 revised 1985). Human recombinant HMGB1 (rhHMGB1) was purchased from Abcam. JNK inhibitor SP600125 was purchased from Selleck.

### Cell culture

The human HCC cell line HepG2, HCCLM3, Huh7, SMMC7721 and MHCC97H was achieved from the Cell Bank of the Chinese Academy of Sciences (Shanghai, China). HCC line cells were all cultured in 4.5g/L glucose DMEM containing 10% FBS (Gbico, USA) and 10% FBS, penicillin (100U/mL), and streptomycin (100μg/mL).

### Q-PCR

Total RNAs of tumor tissues from HCC patients and cells were isolated using Trizol reagent (Life Technology). 2μg of total RNA were reverse-transcribed (Takara). The specific primers used to amplify relevant genes are shown in Supplementary Table 1. The PCR was carried out in triplicate using SYBR Green real-time PCR master mix (Takara) in an ABI. All results are normalized to 18S rRNA expression.

### Immunoblot analysis

Total protein was extracted by lysing cells in RIPA buffer containing protease inhibitor cocktail. Protein samples boiled with 1x loading buffer were separated by sodium dodecyl sulfate polyacrylamide gel electrophoresis (SDS-PAGE) and transferred onto polyvinylidene fluoride (PVDF) membranes. After blocking with 5% BSA in TBS-T, membranes were incubated with the primary antibody at 4° C overnight. Goat-anti-rabbit or mouse IgG conjugated to horseradish peroxidase (HRP) was used as the secondary antibody. Protein was imaged by Tanon system.

### RNA interference

HMGB1 siRNA, Rage siRNA, OCT4 siRNA, miR-21 mimic and miR-21 inhibitor (Riobio) were transfected into cells using lipofectin 2000 according to the manufacturer’s instructions. At the end of the siRNA treatment (48-72 h), the cells were collected for western blot and Q-PCR.

### Immunofluorescence

Immunofluorescence analysis was performed according to protocols. Cells were seeded into 24-well dishes and fixed by 4% paraformaldehyde 24h later. Fixed cells were stained with HMGB1 (Abcam, USA), Rage (Cell Signaling Technology, USA), OCT4 (Cell Signaling Technology, USA) and CD44 (Cell Signaling Technology, USA) followed by FITC-conjugated anti-mouse IgG and Cy3-conjugated anti-rabbit IgG (Abcam, USA). Representative images were detected by fluorescent microscopy (Leica, Germany) and data were processed via ImagePro Plus.

### Immunohistochemistry

Immunohistochemistry of HCC samples were performed as previously described. Briefly, after incubation with CD44 (Cell Signaling Technology, USA), HMGB1 (Abcam, USA), Rage (Cell Signaling Technology, USA), TGFβ1 (Cell Signaling Technology, USA) and OCT4 (Cell Signaling Technology, USA), the sections were stained in an Envision System (DakoCytomation). The negative control is performed without primary antibody. IHC results were scored according to 0, <25%; 1, <50%; 2, <75%; 3, >75% by two experienced pathologists. Data are shown as means ± SEM.

### Sphere formation assay

HepG2 cells (10 cell/μl in SFM) and HCCLM3 cells (10 cell/μl in SFM) were seeded at 100 μl/well in 24-well ultra-low adhesion plates and treated with or without 1ug/ml rhHMGB1 for 7 days. rhHMGB1 was added every other day. The total number of spheres in each well was counted under a microscopy.

### Invasion assays

The invasive ability of HCC cells was measured via 24-well transwell chambers separated by polycarbonate membranes with 8-μm pores and pre-coated with Matrigel. The lower chamber was filled with complete DMEM as a chemo-attractant. Cells in serum-free medium were seeded at 5×10^4^ in the upper chamber and incubated at 37° C in a humidified incubator containing 5% CO2. Cells that migrated to the underside of the membrane were fixed and stained with Giemsa (Sigma, USA), detected, and calculated with a microscope (Leica, Germany).

### Metastasis model *in vivo*

5 x 10^5^ HepG2 cells were transfected with miR-21 negative or inhibitor and then treated with or without rhHMGB1 (1μg/ml) for 24h. Next, those HepG2 cells were collected and injected into nude mice via tail veil. 14 days after injection, livers and lungs were obtained from those mice and colonies were analyzed.

### TGF-β1 ELISA assays

TGF-β1 concentration was measured in the cultural medium according to manuscript instruction by using Elisa Assay (MultiSciences, China).

### Wound healing assays

Cell migration capacity was measured by wound healing assays. The 200μl pipette tip was used to scratch a straight wound in cells seeded in 6-well plates and then cultured in serum-free medium for 24 hours. Images of the wound width were then captured at 0 and 24 hour.

### Statistical analysis

Fisher’s exact tests and χ2 tests were used to determine clinicopathological correlations. The Student’s t-test was used for comparison between variables. GraphPad Prism 6 was used for all statistical analyses. P < 0.05 was considered statistically significant.

## Supplementary Material

Supplementary Figures

Supplementary Table 1
