# Supplementary material for: Extracellular HMGB1 promotes CD44 expression in hepatocellular carcinoma via regulating miR-21
Source: Aging (Albany NY). 2021 Mar 4;13(6):8380–95. doi: 10.18632/aging.202649 (PMC8034936; doi:10.18632/aging.202649)
Supplement: Supplementary Figures [file aging-13-202649-s001.pdf]

SUPPLEMENTARY FIGURES

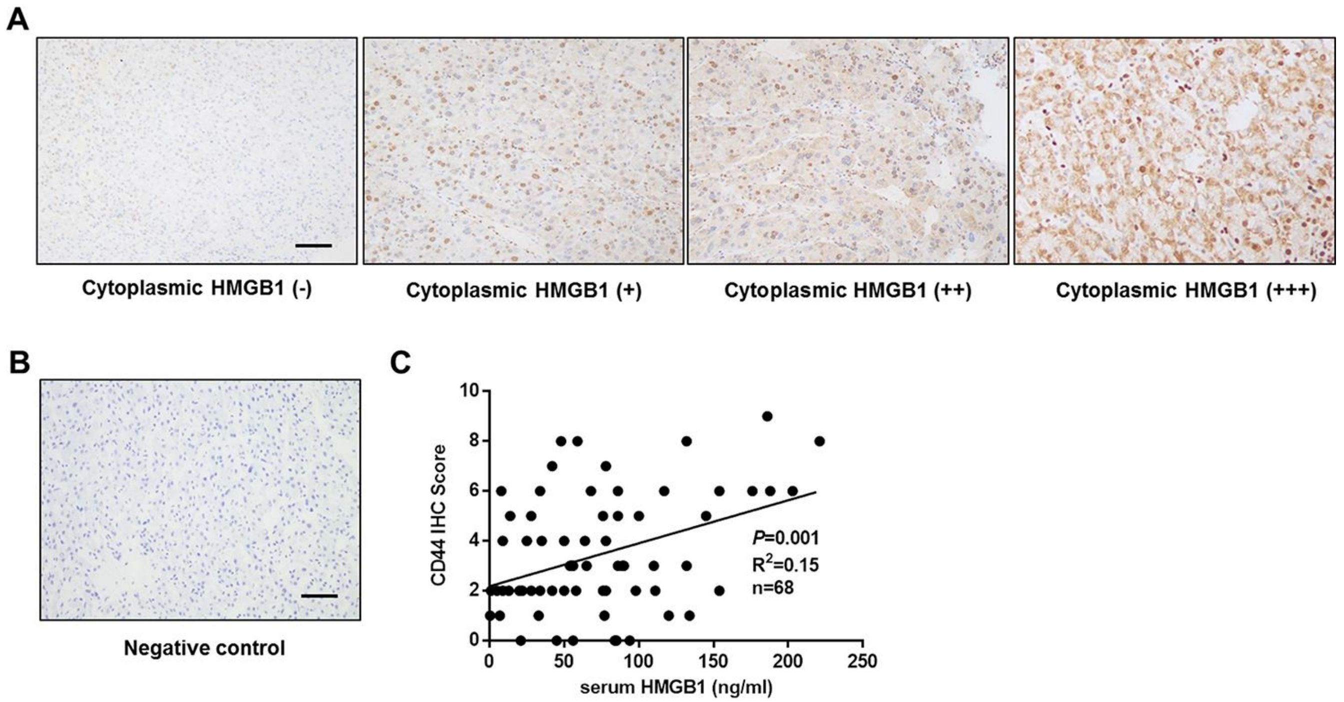

Supplementary Figure 1. Extracellular HMGB1 is correlated with CD44 in HCC. (**A**) Representative images show different intensities of staining cytoplasmic HMGB1 in HCC samples. Scale bars, 100um. (**B**) The negative control image is taken from an identical assay without primary antibody. (**C**) Positive correlation between serum HMGB1 and CD44 IHC score.

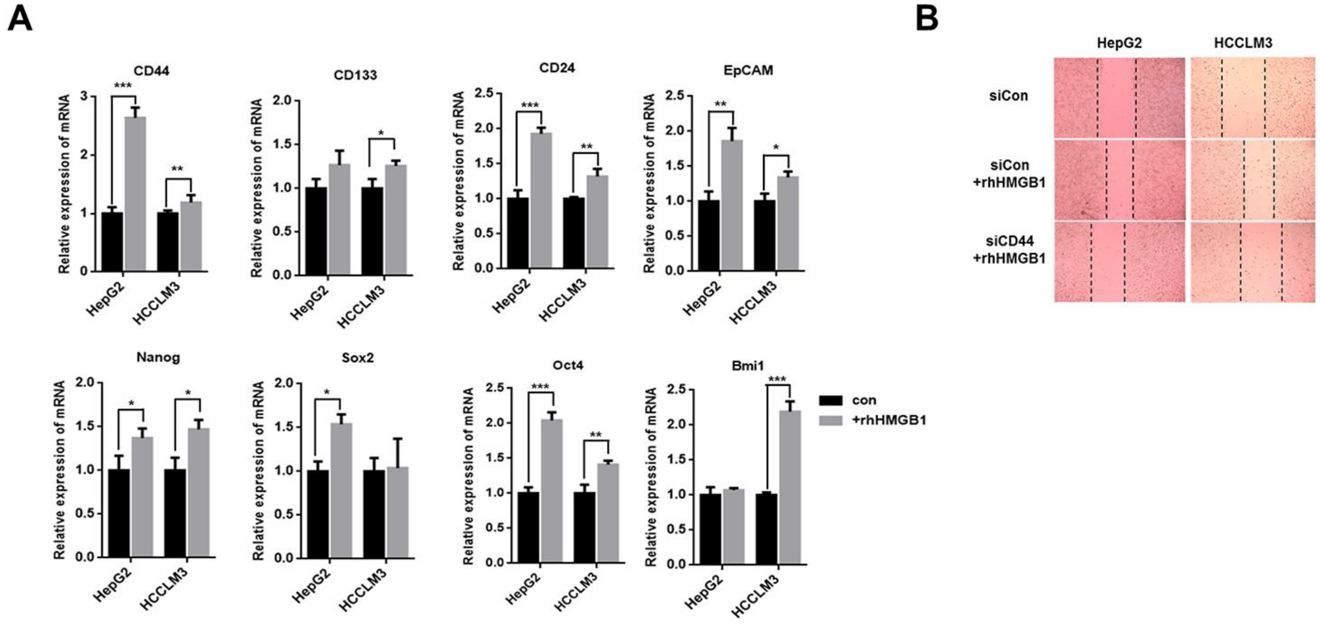

Supplementary Figure 2. Extracellular HMGB1 promotes CSCs formation. (**A**) Q-PCR analysis shows that rhHMGB1 increases the expression of CSCs markers. HepG2 and HCCLM3 cells were cultured with rhHMGB1 (1μg/ml) for 24h. (**B**) Wound healing experiments were performed and results indicate rhHMGB1 promotes the migration of HCC in CD44-dependent way. HepG2 and HCCLM3 cells were transfected with negative siRNA or CD44 siRNA and then cultured with rhHMGB1 (1μg/ml) for 24h. Data are means ± SEM, * means p<0.05, ** means p<0.01, *** means p<0.001 by unpaired student T test.

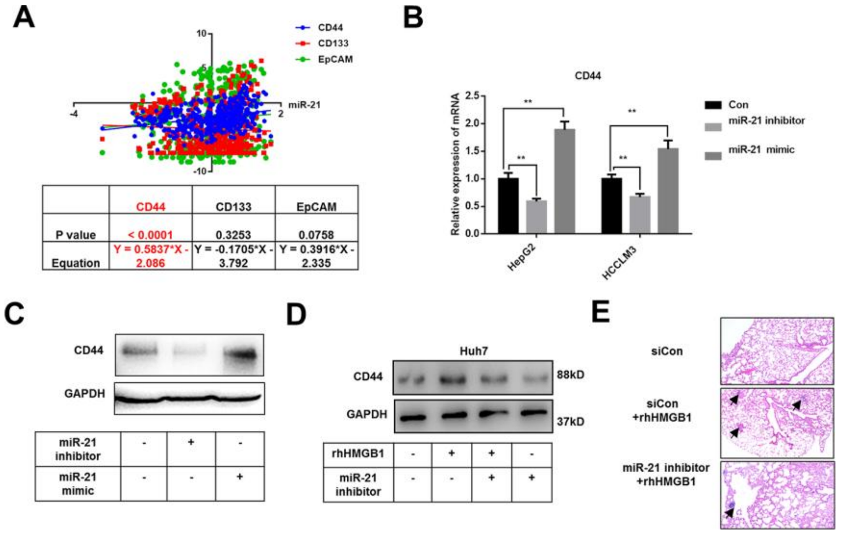

Supplementary Figure 3. miR-21 increases CD44 expression in HCC. (**A**) miR-21 is positively associated with CD44 expression. Data were extracted from TCGA database (**http://xena.ucsc.edu/**). (**B**) Q-PCR analysis shows miR-21 increases CD44 expression. HepG2 and HCCLM3 were transfected with miR-21 mimic or inhibitor respectively. (**C**) Immunoblot analysis indicates that miR-21 upregulates CD44 expression. (**D**) Immunoblot analysis shows that miR-21 inhibitor represses CD44 expression caused by rhHMGB1. Huh7 cells were transfected with negative control or miR-21 inhibitor and then cultured with rhHMGB1 (1μg/ml) for 24h. (**E**) Results from metastasis model *in vivo* show that miR-21 inhibitor represses lung metastasis caused by rhHMGB1. HepG2 cells were transfected with negative control or miR-21 inhibitor and then cultured with rhHMGB1 (1μg/ml) for 24h. Data are means ± SEM, * means p<0.05, ** means p<0.01, *** means p<0.001 by unpaired student T test.

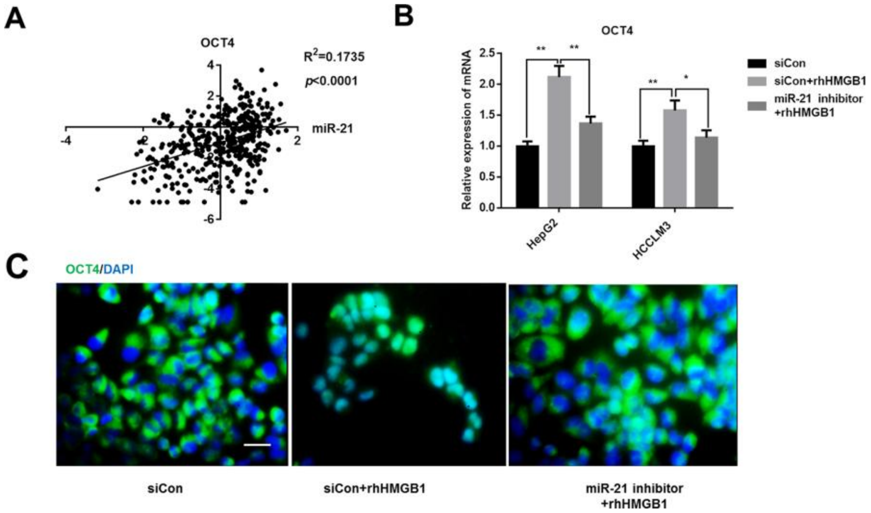

Supplementary Figure 4. miR-21 is essential for extracellular HMGB1-mediated OCT4 expression. (**A**) miR-21 is positively associated with OCT4 expression. Data were extracted from TCGA database (http://xena.ucsc.edu/). (**B**) Q-PCR analysis shows miR-21 inhibitor decreases OCT4 expression caused by rhHMGB1. HepG2 and HCCLM3 cells were transfected with negative control or miR-21 inhibitor and then cultured with rhHMGB1 (1μg/ml) for 24h. (**C**) Results from immunofluorescences staining OCT4 reveal that miR-21 inhibitor restricts OCT4 nuclear translocation caused by rhHMGB1. HepG2 cells were transfected with negative control or miR-21 inhibitor and then cultured with rhHMGB1 (1μg/ml) for 24h. Data are means ± SEM, * means p<0.05, ** means p<0.01, *** means p<0.001 by unpaired student T test.

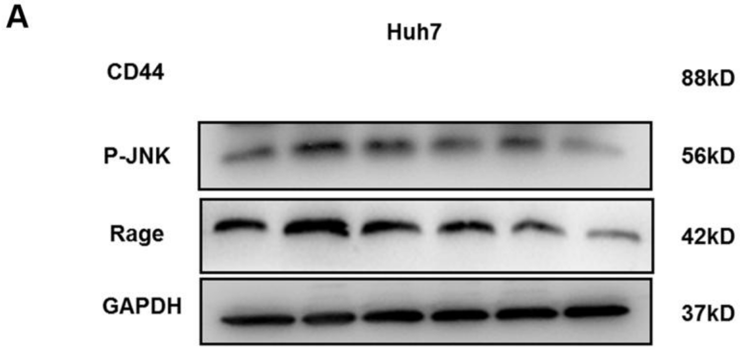

**rhHMGB1**	-	+	+	-	+	-
**SP**	-	-	+	+	-	-
**siCon**	+	+	+	+	-	-
**siRage**	-	-	-	-	+	+

Supplementary Figure 5. Extracellular HMGB1 upregulates CD44 expression via activating miR-21/Rage/JNK signaling in Huh7 cells. (**A**) Immunoblot analysis indicates rhHMGB1 promotes CD44 expression by activating Rage/JNK signaling pathway. Huh7 cells were treated with negative control, Rage siRNA, JNK inhibitor(SP600125,SP,20μm) and rhHMGB1 (1μg/ml) for 24h.
