# Supplementary material for: Extracellular HMGB1 promotes CD44 expression in hepatocellular carcinoma via regulating miR-21
Source: Aging (Albany NY). 2021 Mar 4;13(6):8380–95. doi: 10.18632/aging.202649 (PMC8034936; doi:10.18632/aging.202649)
Supplement: Supplementary Table 1 [file aging-13-202649-s002.pdf]

SUPPLEMENTARY TABLE

Supplementary Table 1. qPCR primer sequences.

Gene	Forward	Reverse
18s	CGGCG ACGACCCATTCGAAC	GAATCGAACC CTGAT TCCCC GTC
Hmgb1	ATA TGG CAA AAG CGG ACA AG	GCA ACA TCA CCA ATG GAC AG
CD44	GGTGAACAAGGAGTCGTC	TTCCAAGATAATGGTGTAGGTG
CD133	AACGGCACCATTGGTCTCTG	AGGAAGGGAGGGAGTCATCC
Epcam	GGGGAACAACTGGATCTGGA	CCAGCAACAACTGCTATCACC
Oct4	CTTGCTGCAGAAGTGGGTGGA	CTGCAGTGTGGGTTTCGGGCA
Nanog	GATTTGTGGGCCTGAAGAAA	TTGGGACTGGTGGAAGAATC
Bim1	GTGAGGAAACTGTGGATGAGGA	TGGAGAAGGAATGGTCCACTTC
Sox2	GCGAACCATCTCTGTGGTCT	GGAAAGTTGGGATCGAACAA
CD24	CTCCTACCCACGCAGATTTATTC	AGAGTGAGACCACGAAGAGAC
TGFβ1	GGCCAGATCCTGTCCAAGC	GTGGGTTTCCACCATTAGCAC
miR-21-5p	TAGCTTATCAGACTGATG	ACATCGAGTGTAGCATA
